# Item and response-category functioning of the Persian version of the KIDSCREEN-27: Rasch partial credit model

**DOI:** 10.1186/1477-7525-10-127

**Published:** 2012-10-18

**Authors:** Peyman Jafari, Zahra Bagheri, Mozhgan Safe

**Affiliations:** 1 Department of Biostatistics, Shiraz University of Medical Sciences, Shiraz, Iran; 2 Fasa University of Medical Sciences, Fasa, Iran

**Keywords:** Quality of life, KIDSCREEN-27, Iran, Rasch model

## Abstract

**Background:**

The purpose of the study was to determine whether the Persian version of the KIDSCREEN-27 has the optimal number of response category to measure health-related quality of life (HRQoL) in children and adolescents. Moreover, we aimed to determine if all the items contributed adequately to their own domain.

**Findings:**

The Persian version of the KIDSCREEN-27 was completed by 1083 school children and 1070 of their parents. The Rasch partial credit model (PCM) was used to investigate item statistics and ordering of response categories. The PCM showed that no item was misfitting. The PCM also revealed that, successive response categories for all items were located in the expected order except for category 1 in self- and proxy-reports.

**Conclusions:**

Although Rasch analysis confirms that all the items belong to their own underlying construct, response categories should be reorganized and evaluated in further studies, especially in children with chronic conditions.

## Findings

The classical test theory (CTT) and the item response theory (IRT) are the two most common methods used to test the reliability and validity of the quality of life instruments. The advantages of IRT models outnumber those of CTT methods [1,2]. While the CTT approach allocates an equal weight to all the items in the instrument and focuses on assessing summated scale scores, IRT models are able to analyze the properties of items individually with respect to the amount of information they provide on the underlying construct [3].

However, the researchers using IRT models are faced with different problems. These models require two crucial assumptions including unidimensionality and local independence to estimate the model parameters. Moreover, model fit indices depend on a variety of factors, including the number of response options and the spread of responses across categories. IRT models also need a huge sample size to guarantee accurate item parameter estimates [1,2,4].

The KIDSCREEN is an international instrument for measuring HRQoL in children and adolescents, which has been simultaneously applied and evaluated in several European countries [5-7]. Structural validity of the KIDSCREEN-27 has been assessed in 13 European countries using CTT and IRT methods [5,8]. Although these studies revealed that all the items fit the data well, none of them discussed the optimal number of response categories except the handbook of the KIDSCREEN questionnaires [9]. The main objective of the current study, hence, was to determine whether the adjacent response categories for each item in the Persian version of the KIDSCREEN-27 were located in the expected order. In the current research, the PCM was used to report item properties and rating scale structure of the KIDSCREEN-27.

## Methods

The target population was Iranian school children aged 8–18 and their parents who were randomly selected by a two-stage cluster random sampling technique from the four educational districts of Shiraz, southern Iran. Written informed consent was obtained from the participants prior to enrollment in the study. The study was approved by the ethical committee of our institution, Shiraz University of Medical Sciences. The Persian version of the KIDSCREEN-27, which was previously translated by the KIDSCREEN group, was filled in by 1083 school children (55.4% boys, 44.6% girls) and 1070 of their parents. The mean (± standard deviation) age of boys and girls was 13.65±2.11 and 12.7±2.65, respectively. It encompasses 27 items divided into five domains including physical well-being (5 items), psychological well-being (7 items), autonomy and parent relation (7 items), social supports and peers (4 items), and school environment (4 items). The participants responded to the items on a 5-point Likert scale from 1=never to 5= always or from 1=not at all to 5=extremely. For ease of interpretation, rating scale categories of negatively worded items were reversed such that higher scores indicated better HRQoL.

Internal consistency for each domain was assessed by Choronbach’s alpha coefficient.

The value of a correlation coefficient of greater than 0.40 between an item and its own domain was considered as an adequate evidence of convergent validity. Discriminant validity was supported whenever a correlation between an item and its hypothesized domain was higher than that with the other scales [10].

The essential assumption of IRT models, unidimensionality, was examined using the Rasch PCM. Moreover, the PCM was used to assess item statistics and response-categories functioning [11,12]. Parameters for this model were estimated using the program WINSTEP [13]. The two key indicators including infit and outfit statistics were used to evaluate whether all the items contribute effectively to their own domain. The range of acceptable values for both infit and outfit item statistics was from 0.7 to 1.3 and values close to 1 were ideal [3]. Items with lower fit statistics were considered redundant and those with high item-fit statistics indicated that the items may not be sufficiently related to the rest of the scale and unidimensionality may not hold [3,11]. Average measures, step calibrations and fit statistics were used to test whether the response categories behaved sufficiently well [3,13]. The categories were considered as misfitting if infit or outfit statistics were greater than 1.5 or less than 0.5 [13]. For the five categories, there are four step calibrations corresponding to the locations on the domain at which participants are able to choose higher as compared lower responses (2 over 1, 3 over 2, 4 over 3, and 5 over 4). Average measures and step calibrations are expected to increase with increasing response categories. The violation of this pattern indicates that the response categories are disordered. In addition to average measure and step calibration estimates, category fit indices and category probability curves (CPC) provide additional information about functioning of response categories. According to Linacre’s criteria [14], categories with an outfit of greater than 2 were considered to be misfit.

## Results

**Figure 1:**
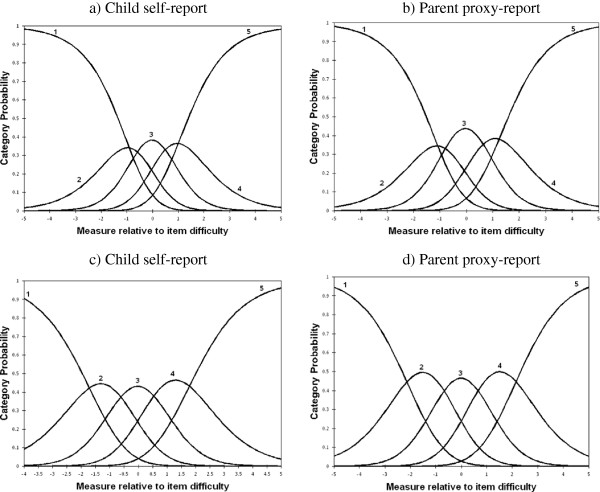
Category probability curves of five response categories for item 6 in the psychological well-being domain and item 2 in the autonomy and parent relation domain in the KIDSCREEN-27.

Tables 1 and 2 represent item difficulty, average measures, step calibrations, and item and category fit indices for self- and proxy-reports. All of the items in the KIDSCREEN-27 demonstrated acceptable infit and outfit statistics (0.7-1.3). Hence, all domains in both self- and proxy-reports can be considered sufficiently unidimensional. Item difficulty estimates ranged from −0.77 to 0.50 and −0.55 to 0.55 for self- and proxy-reports respectively. Items 1 and 4 in the social support and peers domain for child self-report, and items 2 and 4 in the autonomy and parent relation domain for parent proxy-report were the most and least difficult items, respectively. As shown in Tables 1 and 2, the infit and outfit statistics for all response categories, except for “never or not at all”, were within the acceptable range (0.5–1.5). In the child self-report, items 1 and 2 in the physical well-being, items 6 and 7 in the psychological well-being, items 3 and 4 in the autonomy and parent relation, item 3 in the social support and peers, and item 4 in the school environment domains had infit and/or outfit greater than 1.5. Moreover, items 1 and 2 in the physical well-being, items 3 and 6 in the psychological well-being, and item 7 in the autonomy and parent relation domains, in parent-proxy report, had infit and/or outfit greater than 1.5. Within each item, the average measures and step calibrations increased monotonically as the rating scales moved from lower to higher categories. These results correspond to the intersections in the CPC, Figure 1.

**Table 1:** Item and category fit indices for self child-report in the KIDSCREEN-27

	**Item indices**			**Category fit indices**				
	**Difficulty**	**Infit**	**Outfit**	**Average measure, step calibration, infit, outfit**				
**Physical well-being**				1	2	3	4	5
1. how would you say your health is	−0.42	1.01	1.01	−0.92, none, 1.52, 1.81	−0.35, -1.74, 1.05, 1.12	0.32, -0.77, 0.86, 0.81	1.15, 0.74, 0.97, 1.03	2.01, 1.71, 1.03, 1.02
2. Felt fit and well	0.12	1.12	1.09	−0.25, none, 1.87, 2.15	0.04, -1.17, 1.32, 1.43	0.33, -1.03, 0.82, 0.74	1.33, 0.37, 0.87, 0.97	2.22, 1.82, 0.97, 0.97
3. Been physically active	0.39	0.93	0.95	−0.58, none, 1.05, 1.05	−0.02, 1.04, 0.72, 0.65	0.74, -0.71, 1.01, 1.00	1.41, 0.60, 0.89, 0.95	2.20, 1.15, 0.95, 0.98
4. Been able to run well	−0.10	0.87	0.87	−1.00, none, 0.90, 0.92	−0.38, -2.00, 0.66, 0.64	0.62, -0.41, 0.96, 0.89	1.30, 0.87, 0.83, 0.97	2.14, 1.54, 0.97, 0.98
5. Felt full of energy	0.00	1.04	1.05	−0.94, none, 1.16, 1.24	−0.44, -1.53, 0.86, 0.86	0.54, -1.54, 0.86, 0.93	1.52, 0.66, 0.86, 0.27	2.00, 2.41, 1.27, 1.21
**Psychological well-being**								
1. Your life been enjoyable	−0.34	0.85	0.87	−1.70, none, 0.69, 0.65	−0.70, -1.30, 0.65, 0.59	0.21, -1.18, 0.78, 0.71	1.04, 0.64 ,0.86, 1.13	1.81, 1.57, 1.05, 1.03
2. Been in a good mood	−0.33	0.82	0.81	−1.77, none, 0.79, 0.79	−0.61, -2.18, 0.83, 0.80	0.24, -0.59, 0.80, 0.76	1.06, 0.53, 0.77, 0.73	2.12, 2.25, 0.88, 0.91
3. Had fun	0.02	1.22	1.25	−0.60, none, 1.79, 1.93	−0.26, -2.07, 1.12, 1.20	0.48, -0.64, 1.12, 1.18	1.17, 0.57, 1.17, 1.20	2.03, 2.14, 1.17, 1.13
4. Felt sad	0.35	0.93	0.95	−1.13, none, 1.04, 1.09	−0.37, -1.68, -0.97, 1.03	0.66, -0.97, 0.99, 1.03	1.26, 0.22, 0.96, 0.98	2.34, 2.44, 0.96, 0.97
5. Felt so bad that you didn’t want to do anything	0.20	1.14	1.13	−0.09, none, 1.41, 1.39	−0.25, -2.14, 0.91, 0.90	0.70, -0.70, 1.15, 1.12	1.23, 0.87, 1.15, 1.15	2.15, 1.97, 1.22, 1.20
6. Felt lonely	0.20	1.17	1.22	−0.69, none, 1.48, 1.68	−0.12, -0.90, 0.91, 0.95	0.57, -0.59, 1.11, 1.27	0.95, 0.50, 1.30, 1.12	1.94, 1.00, 1.07, 1.11
7. Been happy with the way you are	−0.10	0.84	0.80	−1.26, none, 1.06, 1.21	−0.31, -1.14, 0.87, 0.80	0.17, -0.38, 0.69, 1.60	0.99, 0.18, 0.64, 0.54	2.04, 1.59, 0.90, 0.95
**Autonomy and parent relation**								
1. Had enough time for yourself	0.41	1.11	1.08	−0.29, none, 1.06, 1.21	0.11, -1.77, 1.03, 1.03	0.66, -0.52, 1.02, 1.00	1.41, 0.70, 0.87, 0.88	1.98, 1.59, 1.26, 1.23
2. Been able to do thing	0.50	1.09	1.07	−0.31, none, 1.35, 1.33	0.14, -1.71, 0.96, 0.93	0.61, -0.61, 0.93, 0.85	1.56, 0.54, 0.77, 0.84	1.91, 1.78, 1.37, 1.32
3. Your parent had enough time for you	−0.18	0.84	0.80	−1.04, none, 0.74, 0.69	−0.14, -0.97, 0.95, 0.89	0.22, -0.26, 0.76, 0.59	0.93, 0.12, 0.76, 0.78	1.73, 1.11, 0.91, 0.94
4. Your parent treated you fairly	−0.77	0.83	0.76	−1.59, none, 0.70, 0.79	−0.57, -1.08, 0.87, 0.72	−0.05, -0.38, 0.76, 0.77	0.71, 0.38. 0.81, 0.77	1.53, 1.08, 0.91, 0.95
5. Been able to talk to your parent	−0.10	0.97	1.05	−0.84, none, 0.96, 1.01	−0.21, -0.82, 0.85, 0.78	0.37, -0.49, 0.91, 0.92	1.11, 0.31, 0.94, 1.43	1.61, 1.00, 1.08, 1.07
6. Had enough money to do things as your friend	0.28	1.08	1.05	−0.52, none, 1.11, 1.12	0.05, -0.94, 0.98, 1.03	0.62, -0.48, 0.95, 0.92	1.21, 0.30, 0.88, 0.91	1.79, 1.12, 1.28, 1.20
7. Had enough money for your expenses	−0.14	1.06	1.06	−0.67, none, 1.26, 1.55	−0.15, -1.02, 1.02, 1.17	0.38, -0.30, 0.93, 0.91	0.96, 0.32, 0.83, 0.79	1.67, 0.99, 1.14, 1.09
**Social support and peers**								
1. Spent time with your friends	0.30	1.10	1.09	−0.91, none, 1.42, 1.46	−0.49, -2.25, 0.89, 0.89	0.53, -0.97, 0.98, 0.97	1.55, 0.55, 0.99, 1.02	2.33, 2.66, 1.33, 1.23
2. Had fun with your friends	−0.20	0.83	0.83	−1.56, none, 0.96, 0.98	−0.81, -1.66, 0.74, 0.70	0.10, -1.03, 0.71, 0.65	1.24, 0.43, 0.71, 0.80	2.22, 2.26, 1.02, 1.00
3. You and your friends helped each other	−0.53	0.96	0.94	−2.01, none, 0.70, 0.70	−0.86, -2.15, 0.88, 0.86	0.12, -1.04, 0.86, 0.83	1.15, 0.94, 0.85, 0.84	2.09, 2.24, 1.21, 1.18
4. Been able to rely on your friends	−0.43	1.08	1.07	−0.95, none, 1.21, 1.18	−0.21, -1.92, 0.94, 0.92	0.60, -0.56, 1.00, 0.97	1.59, 0.56, 0.87, 0.96	2.29, 1.92, 1.34, 1.29
**School environment**								
1. Been happy at school	0.27	1.05	1.03	−0.92, none, 1.01, 1.02	−0.08, -1.66, 1.10, 1.12	0.63, -1.29, 0.92, 0.88	1.55, 0.77, 1.03, 1.03	2.44, 2.18, 1.15, 1.11
2. Got on well at school	0.21	0.89	0.89	−0.97, none, 1.03, 1,03	−0.44, -1.87, 0.78, 0.77	0.62, -1.52, 0.82, 0.79	1.66, 1.06, 0.79, 0.83	2.55, 2.33, 1.02, 1,03
3. Been able to pay attention	−0.09	0.94	0.92	−0.99, none, 1.14, 1.14	−0.36, -2.51, 0.96, 0.95	0.40, -1.02, 0.75, 0.71	1.54, 0.69, 0.91, 0.93	2.48, 2.83, 1.05, 1.03
4. Got along well with your teachers	−0.39	1.09	1.18	−1.06, none, 1.15, 1.37	−0.50, -1.01, 0.92, 0.97	0.24, -0.75, 0.99, 1.10	1.13, 0.20, 0.97, 1.26	1.92, 1.57, 1.29, 1.18

**Table 2:** Item and category fit indices for parent proxy-report in the KIDSCREEN-27

	**Item indices**			**Category fit indices**				
	**difficulty**	**Infit**	**Outfit**	**Average measure, step calibration, infit, outfit**				
**Physical well-being**				1	2	3	4	5
1. how would you say your health is	−0.32	1.22	1.21	−1.18, none, 1.55, 1.58	−0.16, -3.01, 1.45, 1.53	0.55, -0.79, 0.99, 0.98	1.70, 1.10, 1.01, 1.04	2.64, 2.70, 1.37, 1.33
2. Felt fit and well	−0.45	0.95	0.93	−0.82, none, 1.90, 2.20	−0.60, -2.01, 1.19, 1.20	0.24, -1.91, 0.85, 0.83	1.55, 0.90, 0.81, 0.77	2.89, 3.02, 0.91, 0.92
3. Been physically active	0.47	0.91	0.90	−0.87, none, 1.17, 1.16	−0.15, -1.69, 0.82, 0.80	0.83, -0.81, 0.81, 0.72	1.85, 0.78, 0.89, 1.00	2.84, 1.71, 0.93, 0.94
4. Been able to run well	0.29	0.89	0.90	−1.13, none, 0.91, 0.92	−0.34, -2.03, 0.90, 0.96	0.75, -1.04, 0.80, 0.76	1.83, 0.62, 0.90, 0.94	2.93, 2.45, 0.95, 0.96
5. Felt full of energy	0.01	0.99	1.00	−1.43, none, 1.10, 1.21	−0.79, -1.87, 0.82, 0.80	0.49, -1.77, 0.83, 0.81	1.88, 0.54, 0.89, 0.99	2.74, 3.10, 1.26, 1.19
**Psychological well-being**								
1. Your life been enjoyable	−0.24	0.87	0.88	−1.64, none, 0.75, 0.73	−0.59, -1.76, 0.81, 0.78	0.32, -1.19, 0.82, 0.89	1.27, 0.80, 0.86, 0.91	2.16, 2.15, 0.99, 0.99
2. Been in a good mood	−0.23	0.79	0.78	−1.43, none, 1.00, 1.00	−0.77, -1.43, 0.70, 0.65	0.15, -1.26, 0.68, 0.63	1.18, 0.29, 0.75, 0.77	2.27, 2.49, 0.88, 0.91
3. Had fun	0.00	1.10	1.12	−1.26, none, 1.16, 1.28	−0.24, -2.17, 0.99, 1.01	0.51, -1.01, 1.07, 1.04	1.43, 0.78, 1.00, 1.11	2.11, 2.41, 1.26, 1.21
4. Felt sad	0.24	1.03	1.02	−0.95, none, 1.27, 1.37	−0.49, -1.76, 0.77, 0.78	0.66, -1.53, 0.93, 0.93	1.37, 0.77, 1.07, 1.02	2.48, 2.53, 1.11, 1.08
5. Felt so bad that you didn’t want to do anything	0.39	1.13	1.13	−0.70, none, 1.41, 1.45	−0.12, -2.01, 1.02, 1.04	0.79, -1.11, 1.14, 1.12	1.32, 0.96, 1.27, 1.25	2.54, 2.16, 0.97, 0.97
6. Felt lonely	−0.03	1.11	1.11	−0.73, none, 1.61, 1.71	−0.28, -1.08, 1.05, 1.11	0.43, -0.80, 0.95, 1.03	1.02, 0.64, 1.14, 1.03	2.03, 1.24, 0.98, 0.99
7. Been happy with the way you are	−0.11	0.93	0.93	−1.25, none, 1.61, 1.32	−0.52, -1.49, 0.81, 0.76	0.15, -1.09, 0.77, 0.71	1.32, 0.12, 0.72, 0.88	2.11, 2.46, 1.16, 1.09
**Autonomy and parent relation**								
1. Had enough time for yourself	−0.20	1.15	1.12	−0.64, none, 1.17,1.20	0.07, -1.58, 1.23, 1.35	0.47, -0.59, 1.02, 1.02	1.12, 0.15, 1.02, 1.00	1.80, 1.99, 1.25, 1.13
2. Been able to do thing	−0.02	1.06	1.05	−0.77, none, 1.06, 1.06	0.07, -2.31, 1.08, 1.08	0.60, -0.67, 0.95, 0.95	1.32, 0.66, 0.93, 0.93	1.95, 2.15, 1.19, 1.14
3. Your parent had enough time for you	0.27	0.95	0.94	−0.27, none, 1.41, 1.59	−0.06, -1.53, 0.73, 0.67	0.58, -0.69, 0.79, 0.71	1.37, 0.26, 0.81, 0.90	2.06, 1.95, 1.07, 1.05
4. Your parent treated you fairly	−0.16	0.91	0.92	−0.46, none, 1.40, 1.69	−0.31, -1.01, 0.79, 0.80	0.35, -0.75, 0.81, 0.78	1.08, 0.22, 0.74, 0.75	1.89, 1.63, 0.93, 0.95
5. Been able to talk to your parent	−0.21	0.98	1.04	−0.74, none, 1.11, 1.22	−0.14, -1.21, 0.91, 0.98	0.38, -0.38, 0.85, 0.75	1.11, 0.20, 0.95, 1.27	1.73, 1.39, 1.06, 1.06
6. Had enough money to do things as your friend	0.20	1.02	1.05	−0.44, none, 1.21, 1.34	0.10, -1.26, 0.98, 1.04	0.66, -0.51, 0.99, 1.05	1.18, 0.34, 0.94, 0.95	1.95, 1.42, 1.01, 1.00
7. Had enough money for your expenses	0.12	0.90	0.87	−0.58, none, 1.05, 1.16	−0.04, -1.21, 0.79, 0.73	0.53, -0.41, 0.81, 0.70	1.22, 0.22, 0.77, 0.76	1.91, 1.40, 1.03, 1.01
**Social support and peers**								
1. Spent time with your friends	0.55	1.06	1.06	−1.52, none, 1.31, 1.32	−0.51, -2.62, 0.95, 0.97	0.62, -1.06, 0.99, 0.97	1.73, 0.85, 0.91, 0.92	2.67, 2.82, 1.25, 1.23
2. Had fun with your friends	0.36	1.15	1.16	−2.57, none, 0.74, 0.76	−1.06, -2.65, 0.82, 0.81	0.03, -0.99, 0.72, 0.69	1.28, 0.92, 0.70, 0.71	2.54, 2.73, 0.85, 0.88
3. You and your friends helped each other	−0.36	0.77	0.77	−2.09, none, 1.52, 1.53	−1.20, -2.92, 0.83, 0.82	0.03, -0.89, 0.83, 0.77	1.25, 1.00, 0.87, 0.88	2.27, 2.82, 1.25, 1.22
4. Been able to rely on your friends	−0.55	1.00	0.97	−1.52, none, 1.38, 1.40	−0.54, -2.54, 1.03, 1.07	0.50, -0.90, 1.09, 1.12	1.52, 0.60, 1.10, 1.09	2.55, 2.84, 1.27, 1.20
**School environment**								
1. Been happy at school	0.12	1.09	1.08	−1.01, none, 1.24, 1.31	−0.53, -2.14, 0.88, 0.86	0.61, -1.64, 1.04, 1.03	1.81, 0.85, 0.98, 1.01	2.73, 2.93, 1.30, 1.24
2. Got on well at school	0.41	0.84	0.84	−1.23, none, 0.83, 0.79	−0.42, -2.20, 0.85, 0.85	0.69, -1.42, 0.72, 0.70	2.01, 0.90, 0.73, 0.74	2.99, 2.72, 1.06, 1.05
3. Been able to pay attention	0.02	0.86	0.86	−1.58, none, 0.78, 0.79	−0.60, -2.92, 0.73, 0.69	0.52, -0.86, 0.74, 0.70	1.85, 0.61, 0.87, 0.91	2.84, 3.17, 1.10, 1.08
4. Got along well with your teachers	−0.55	1.21	1.22	−0.99, none, 1.59, 2.19	−0.52, -1.42, 1.23, 1.46	0.14, -0.85, 1.08, 1.19	1.13, 0.13, 0.99, 1.05	2.30, 2.14, 1.31, 1.15

Table 3 shows that all the domains have adequate internal consistency (greater than 0.7). Moreover, scaling success rates for convergent and discriminant validity were 100% in all domains.

**Table 3:** Cronbach’s alpha coefficient, convergent and discriminant validity for the KIDSCREEN-27 and score domains for the Iranian school children

					**Convergent validity**^**a**^		**Discriminant validity**^**b**^	
**Scale**	**No. items**	**n**	**mean±SD**	**α**	**Range of correlation**	**Scaling success (percent)**	**Range of correlation**	**Scaling success (percent)**
**Child self-report**								
Physical well-being	5	1083	51.65±11.32	0.79	0.62-0.76	5/5(100)	0.25-0.48	20/20(100)
Psychological well-being	7	1083	44.76±10.90	0.85	0.64-0.74	7/7(100)	0.16-0.57	28/28(100)
Autonomy and parent relation	7	1083	48.61±10.60	0.83	0.64-0.69	7/7(100)	0.27-0.51	28/28(100)
Social support and peers	4	1083	44.66±9.40	0.75	0.73-0.76	4/4(100)	0.23-0.43	16/16(100)
School environment	4	1083	51.32±9.94	0.73	0.63-0.77	4/4(100)	0.21-0.47	16/16(100)
**Parent proxy-report**								
Physical well-being	5	1070	50.36±10.94	0.81	0.63-0.80	5/5(100)	0.24-0.49	20/20(100)
Psychological well-being	7	1070	44.05±12.22	0.83	0.65-0.74	7/7(100)	0.14-0.57	28/28(100)
Autonomy and parent relation	7	1070	49.25±11.78	0.81	0.59-0.69	7/7(100)	0.19-0.51	28/28(100)
Social support and peers	4	1070	45.67±10.93	0.77	0.73-0.82	4/4(100)	0.23-0.45	16/16(100)
School environment	4	1070	50.49±10.91	0.75	0.66-0.81	4/4(100)	0.22-0.47	16/16(100)

a. Number of correlation between items and hypothesized scale corrected for overlap ≥ 0.4/ total number of convergent validity tests.

b. Number of convergent correlations significantly higher than discriminant correlations/Total number of correlations.

## Discussion

In the current study, Cronbach's alpha coefficients for all five domains conformed to those obtained in the combined sample from all European countries [8]. The Rasch PCM analysis of the self- and proxy-reports showed that no item was misfitting. These findings are in the same line with those of the previous study conducted in 13 European countries, indicating that each of the test items measures the underlying construct adequately [8]. Although average measures and step calibrations for all five response categories increased monotonically, 5 and 8 out of 27 items had category fit statistics greater than 1.5 in the self- and proxy-reports, respectively. According to Linacre [14], for a five category scale, advances of at least 1.0 logits between step calibrations are needed in order to achieve the optimal number of response categories. As seen in Tables 1 and 2, the advance in step calibrations from a rating of 1 to 2 to a rating of 2 to 3 is less than 1.0 logits in almost all items. For example, in item 2 for child self-report, step calibrations advance from 1.52 to 1.05, a distance of 0.47. This is not sufficiently large to meet the criteria. These findings indicate that categories 1 (never or not at all) and 2 (seldom or slightly) should be combined in all items for self- and proxy-reports. Similar results were also observed in the Persian version of the PedsQL™ 4.0 Generic Core Scales [15].

Just as in the case with the PedsQL™ 4.0 on Iranian children with chronic conditions [16,17], this study showed that the Persian version of the KIDSCREEN-27 has a good internal consistency, and excellent convergent and discriminant validity. However, although the PCM showed that all the items contributed adequately to their own domain, Rasch analysis revealed that the number of response categories should be reduced from five to four in the Persian version of the KIDSCREEN-27. It is not clear whether this problem is due to the meaning of the response options in the Persian language or an artifact of a mostly healthy schoolchildren who did not choose the full range of the response scale [15]. Therefore, the response categories should be evaluated in further validation studies, especially in large samples of chronically ill children.

## Abbreviations

HRQoL: Health-related quality of life; PCM: Partial credit model; CTT: Classical test theory; IRT: Item response theory; CPC: Category probability curves; DIF: Differential item functioning; SD: Standard deviation.

## Competing interests

The authors declare that they have no competing interests.

## Authors’ contribution

PJ researched and analyzed the data, and wrote the manuscript, ZB analyzed the data and wrote the manuscript, MS researched and analyzed the data. All authors read and approved the final manuscript.
